# Action of Shiga Toxin Type-2 and Subtilase Cytotoxin on Human Microvascular Endothelial Cells

**DOI:** 10.1371/journal.pone.0070431

**Published:** 2013-07-30

**Authors:** María M. Amaral, Flavia Sacerdoti, Carolina Jancic, Horacio A. Repetto, Adrienne W. Paton, James C. Paton, Cristina Ibarra

**Affiliations:** 1 Laboratorio de Fisiopatogenia, Departamento de Fisiología, Facultad de Medicina, Universidad de Buenos Aires, Buenos Aires, Argentina; 2 Laboratorio de Inmunidad Innata, Instituto de Medicina Experimental (IMEX-CONICET), Academia Nacional de Medicina, Buenos Aires, Argentina; 3 Servicio de Pediatría, Hospital Nacional Profesor Alejandro Posadas, Buenos Aires, Argentina; 4 Research Centre for Infectious Diseases, School of Molecular and Biomedical Science, University of Adelaide, Adelaide, Australia; National Cancer Institute, United States of America

## Abstract

The hemolytic uremic syndrome (HUS) associated with diarrhea is a complication of Shiga toxin (Stx)-producing *Escherichia coli* (STEC) infection. In Argentina, HUS is endemic and responsible for acute and chronic renal failure in children younger than 5 years old. The human kidney is the most affected organ due to the presence of very Stx-sensitive cells, such as microvascular endothelial cells. Recently, Subtilase cytotoxin (SubAB) was proposed as a new toxin that may contribute to HUS pathogenesis, although its action on human glomerular endothelial cells (HGEC) has not been described yet. In this study, we compared the effects of SubAB with those caused by Stx2 on primary cultures of HGEC isolated from fragments of human pediatric renal cortex. HGEC were characterized as endothelial since they expressed von Willebrand factor (VWF) and platelet/endothelial cell adhesion molecule 1 (PECAM-1). HGEC also expressed the globotriaosylceramide (Gb3) receptor for Stx2. Both, Stx2 and SubAB induced swelling and detachment of HGEC and the consequent decrease in cell viability in a time-dependent manner. Preincubation of HGEC with C-9 −a competitive inhibitor of Gb3 synthesis-protected HGEC from Stx2 but not from SubAB cytotoxic effects. Stx2 increased apoptosis in a time-dependent manner while SubAB increased apoptosis at 4 and 6 h but decreased at 24 h. The apoptosis induced by SubAB relative to Stx2 was higher at 4 and 6 h, but lower at 24 h. Furthermore, necrosis caused by Stx2 was significantly higher than that induced by SubAB at all the time points evaluated. Our data provide evidence for the first time how SubAB could cooperate with the development of endothelial damage characteristic of HUS pathogenesis.

## Introduction

Hemolytic uremic syndrome (HUS) is characterized by non-immune hemolytic anemia, thrombocytopenia and acute renal failure [1]. The classical form of HUS is a complication of Shiga toxin (Stx)-producing *Escherichia coli* (STEC) infection, the most prevalent infectious agent responsible for the development of this pathology [2].

In Argentina, HUS is endemic with a high incidence of more than 500 cases per year, being the most common cause of acute renal failure and the second leading cause of chronic renal failure in children younger than 5 years old [3,4].

Clinical and histological renal damage has been strongly associated with Stx types 1 and 2 (Stx1, Stx2) produced by O157:H7 STEC, although strains that only express Stx2 are highly prevalent in Argentina [5]. However, other non-O157:H7 serotypes have been shown to cause HUS [6].

STEC are present in the intestinal tract of healthy cattle and disease outbreaks are frequently due to ingestion of undercooked ground beef, manure-contaminated water, vegetables, fruit or unpasteurized milk.

After bacteria colonize the bowel, Stx is released into the gut lumen and then absorbed into the circulation, where the toxin reaches vascular endothelial cells and finally binds its specific receptor, the globotriaosylceramide (Gb3) [7]. This receptor is located on the plasma membrane of target cells, particularly microvascular endothelial cells present in the kidneys [8], brain and other organs. Nevertheless the human kidney is the most affected organ in diarrhea-associated HUS, the likely reason being the presence of very Stx-sensitive cells which express high amounts of biologically active Stx receptor [9]. Indeed, human microvascular endothelial cells express 50-fold higher Gb3 levels than endothelial cells derived from large vessels [10]. Another reason is that the high volume of blood flow and filtration rate increase the chance of Stx interaction with cells of the renal microvasculature and the filtration barrier [9].

Endothelial dysfunction is essential to the development of microvascular lesions in HUS [11,12] and it is well known that Stx is able to increase and expand kidney injury through favoring interactions between the endothelium and leukocytes [13] and platelets [14]. The characteristic lesion of HUS, thrombotic microangiopathy, consists of thickening of arterioles and capillaries, swelling and detachment of endothelial cells from the basement membrane, and platelet thrombi that obstruct the microcirculation of different organs, predominantly the kidney [15]. This cell damage is induced by different mechanisms, such as inhibition of protein synthesis and increases in the protein levels of chemokines, cytokines, and adhesion molecules [14,16–18]. In addition, Gb3 expression and Stx toxicity are increased by inflammatory cytokines such as TNFα, in human glomerular endothelial cells [19].

The concentration of free Stx in serum of patients with HUS could not be established [2,20] because the toxin binds different types of cells including erythrocytes [21], platelets [22] and monocytes [23]. In addition it has been postulated that Stx circulates bound to polymorphonuclear leukocytes (PMN) [24], so endothelial cells would be exposed to very small amounts of free Stx [25]. Recently, Brigotti et al. [26] suggested that the extent of renal damage in children with HUS could depend on the amount of Stx presented to their PMN, which could then deliver the toxin to the renal endothelium.

Several non-O157 STEC strains produce Subtilase cytotoxin (SubAB), which may contribute to HUS pathogenesis. SubAB was discovered in a strain of STEC belonging to serotype O113:H21 that caused an outbreak of HUS in South Australia [27]; this serotype has also been isolated from children with HUS in Argentina [28]. SubAB action on eukaryotic cells involves highly specific A-subunit-mediated proteolytic cleavage of the endoplasmic reticulum (ER) chaperone BiP (GRP78) [29]. This triggers a massive ER stress response, ultimately leading to apoptosis [30–32]. Recently, it was shown that this toxin increases the tissue factor-dependent factor Xa generation in cultured human umbilical vein endothelial cells and human macrophages, suggesting a direct procoagulant effect [33]. SubAB had a strong preference for binding glycans terminating in *N*-glycolylneuraminic acid (Neu5Gc) [34] and this monosaccharide was postulated as the key component of the SubAB receptor; NeuGc is not synthesized in humans, but can be incorporated into human tissues through dietary intake. Several SubAB-binding proteins containing Neu5Gc were identified in Vero [35] and HeLa cells [36]. So far, the effects of SubAB on human glomerular endothelial cells (HGEC) and the associated mechanism of action have not been described. In this study we have developed primary cultures of HGEC to analyze the effects of SubAB in comparison with those caused by Stx2.

## Materials and Methods

### Reagents

Toxins: Stx2 was provided by Phoenix Laboratory, while SubAB was purified as described previously [29].

Competitive inhibitor of Gb3 synthesis: C-9 [(1R, 2R)-nonanoic acid [2-(2’, 3’-dihydro-benzo [1–4]dioxin-6’-yl)-2-hydroxy-1-pyrrolidin-1-ylmethyl-ethyl]-amide-L-tartaric acid salt] was from Genzyme Corp., USA.

### Cell culture

Human glomerular endothelial cells (HGEC) were isolated from kidneys removed from different pediatric patients undergoing nephrectomies performed at Hospital Nacional “Alejandro Posadas”, Buenos Aires, Argentina (written informed consent was obtained from the next of kin, caretakers, or guardians on the behalf of the minors/children participants involved in our study). The Ethics Committee of the University of Buenos Aires approved the use of human renal tissues for research purposes. The method used for glomerular endothelial cell isolation was adapted from that described by McGinn et al. [37]. Brieﬂy, segments of macroscopically normal human renal cortex were minced using a scalpel blade and enzyme digested using 0.1% collagenase type I in buffer Hanks Balanced Salt Solution (HBSS) with 0.1% fetal calf serum (FCS) at 37°C for 30 min. Enzyme digestion was stopped by the addition of ice cold HBBS containing 5% FCS and the sample was washed twice with cold HBBS containing 5% FCS. The tissue was filtered through a 70 µm Nylon cell strainer. The glomeruli were collected on the top of the strainer, washed with cold HBBS containing 5% FCS and then incubated with 0.6% collagenase type I in HBSS with 0.1% FCS at 37°C for 30 min. Finally, the enzyme digestion was stopped and washed, as described above. The cells were resuspended in complete medium: M199 medium (Sigma, USA) supplemented with 20% FCS, 3.2mM L-glutamine, 100 U/ml penicillin/streptomycin (GIBCO, USA) and 25 µg/ml endothelial cell growth supplement ECGS (Sigma, USA) and then cultured in 0.2% gelatin (BDH, United Kingdom) coated ﬂasks. Cells were grown at 37°C in a humidiﬁed 5% CO_2_ incubator and subcultured at conﬂuence by trypsinization with 0.05% trypsin-0.02% EDTA (GIBCO, USA). For growth-arrested conditions, a medium with a half of FCS concentration (10%) and without ECGS was used. At the experiments, cells were used between 2–7 passages.

### Cell characterization

Flow cytometry: Confluent cells were detached by trypsin and the presence of von Willebrand factor (VWF) was analyzed. Cells were fixed with 4% paraformaldehyde and permeabilized with saponin (0.1% in PBS). Then, cells were incubated with a polyclonal rabbit anti-human VWF (DAKO, Argentina) in PBS with 0.5% FCS and then stained with a goat anti-rabbit FITC. The stained cells were washed with saponin buffer twice. Finally, cells were resuspended in isoflow and then were acquired (approximately 50,000 events) by a FACS flow cytometer. In all cases, isotype matched control antibodies were used, and a gate based on the forward and orthogonal light scatter was defined to exclude cell debris. Analysis was performed using Cell Quest software (BD Biosciences, USA). The results are expressed as the mean fluorescence intensity (MIF) relative to control cells that do not express VWF (Ctrl).

Microscopy: Endothelial cells were morphologically characterized at conﬂuence by phase contrast microscopy. Cells were seeded on gelatin coated glass coverslips (12 mm), then washed with PBS at pH 7.4 and fixed for 2 h at room temperature with alcohol 96°, stained with hematoxylin–eosin (H&E) and observed by light microscopy (×200 and ×400, Zeiss Axiophot, Germany). Toxin-treated cells were previously incubated with Stx2 (10 ng/ml) or SubAB (3 µg/ml) for 24 h and then stained with H&E, as described above. Cell counts were performed on four fields of ×200 magnifications each. Cell area values were obtained using ImageJ software (National Institutes of Health, USA).

Platelet/endothelial cell adhesion molecule 1 (PECAM-1) expression was analyzed by confocal microscopy. HGEC seeded on gelatin-coated glass coverslips (12 mm) were washed with PBS at pH 7.4 and fixed with 3% paraformaldehyde (10 min on ice). Fixed cells were directly labeled with a mouse monoclonal anti-human CD31 conjugated with FITC (Sigma, USA). At the optimal antibody concentrations, no background labeling of the nucleus and mitochondria was detected (data not shown). Coverslips were mounted on glass slides using Fluoromount G (SouthernBiotech, USA). Immunofluorescence images were acquired with a FluoView FV1000 confocal microscope (Olympus, Japan) using a Plapon ×60 1.42NA oil immersion objective and images were analyzed using the Olympus FV10-ASW software.

### Neutral red cytotoxicity assay

The neutral red cytotoxicity assay was adapted from previously described protocols [38]. HGEC were plated in gelatin coated 96-well plates and grown to confluence in complete M199 medium. The cells were then washed in PBS at pH 7.4 and exposed to Stx2 (0.001 ng/ml to 100 ng/ml) or SubAB (0.15 ng/ml to 1500 ng/ml) in growth-arrested conditions for 24, 48 and 72 h. For co-treatment assays, cells were incubated with Stx2 and SubAB together, using the same concentrations and times studied in the experiments described with each toxin alone. Then, two hundred microliters of freshly diluted neutral red in M199 was added to a final concentration of 10 μg/ml and cells were incubated for an additional 1 h at 37°C in 5% CO_2_. Cells were then washed with 200μl 1% CaCl_2_ + 1% formaldehyde and solubilized in 200 μl 1% acetic acid in 50% ethanol. Absorption in each well was read in an automated plate spectrophotometer at 540nm. Results were expressed as neutral red uptake percentage, where 100% represents control cells incubated under identical conditions but without toxin treatment. The 50% cytotoxic dose (CD_50_) corresponds to the dilution required to kill 50% of cells. To examine the effect of C-9, cells were incubated in the presence of C-9 (from 0.05 to 50 µM) per 48 h before the addition of the toxins. The cells were then incubated with or without 10 ng/ml of Stx2 or 3 µg/ml of SubAB for additional 24 h. Finally, cell viability was established by neutral red uptake.

### Gb3 expression

Microscopy: Gb3 expression was analyzed by confocal microscopy. HGEC were seeded on gelatin coated glass coverslips (12 mm), then washed with PBS at pH 7.4 and fixed with 3% paraformaldehyde (10 min on ice). Fixed cells were first incubated overnight with a rat anti-human CD77 (AbD Serotec, USA) and then with a goat IgM anti-rat conjugated with FITC, for 2 h. Coverslips were mounted on glass slides using Fluoromount G. Immunofluorescence images were acquired with a FluoView FV1000 confocal microscope (Olympus, Japan) using a Plapon ×60 1.42NA oil immersion objective and images were analyzed using the Olympus FV10-ASW software.

Thin-layer chromatography: Gb3 was detected by thin-layer chromatography (TLC). HGEC were seeded in tissue culture flasks and grown at 37°C in an atmosphere of 5% CO_2_ until the cells were nearly confluent. Adherent cell monolayers were released from the flask by trypsinization as described above, collected by centrifugation, and resuspended in PBS at pH 7.4. Cells were washed twice with PBS at pH 7.4 to deplete the serum lipids. Total HGEC glycolipids were extracted according to the method of Bligh and Dyer [39]. Briefly, 3 ml of chloroform: methanol 2: 1 v/v was added to the cells, and incubated on ice for 15 min. Two ml of chloroform: water (1:1) was added to the tube and centrifugated at 3,000 rpm for 5 min to separate phases. The upper aqueous phase was removed, and the lower phase was brought to dryness. One ml of methanol and 0.1 ml of 1.0 M NaOH was added to the dried residue, and incubated 16 h at 37 °C.

After the addition of 2 ml of chloroform and 0.5 ml water and separation of the phases, the upper phase was removed. The lower phase, corresponding to the neutral glycolipid extract, was brought to dryness and used for Gb3 determination. Fractionated lipids were subjected to TLC with a silica gel 60 aluminum plate previously activated by incubation 15 min at 100°C, in a glass tank with a mixture of chloroform, methanol, and water (65:35:8). A purified glycosphingolipid standard (1-2 µg) (Matreya, USA) was also added to the plate for comparison. After the solvent front reached the top of the plate, the gel matrix was air dried and treated with a solution of orcinol, water and sulfuric acid (Acros Organics, USA) to visualize the separated carbohydrate and glycolipid components. The densitometric analysis of Gb3 bands was analyzed by Image Quant 5.0 software.

### Analysis of Gb3 content in HGEC by an enzymatic fluorometric method

Glycolipid extract was dispersed in 100 μl of 0.15 mol/l acetate buffer at pH 4.5; and sodium taurodeoxycholate (Sigma, USA) was added to emulsify the glycolipids at a final concentration of 0.046 mol/l. Gb3 present in the HGEC glycolipids was hydrolyzed to galactose and lactosylceramide by the addition of 10 μl of 1 mg/ml agalsidase alfa (Shire HGT, United Kingdom) and incubation at 37 °C overnight. Agalsidase alfa is available in a 1 mg/ml solution, and was stored aliquoted at −20°C until use without significant loss of enzymatic activity. To quantify the galactose produced by the enzymatic hydrolysis, 3 mm-filter paper discs were impregnated with the reaction solution. After drying the paper discs at room temperature for 4 h, galactose was determined by a modified enzymatic fluorometric method using galactose dehydrogenase, diaphorase and resazurine. The results were expressed as Gb3 µg/1.10^6^ cells [40].

### Analysis of cell death mechanisms induced by toxins

Microscopy: HGEC were seeded on gelatin coated glass coverslips (12 mm) and then were washed with PBS at pH 7.4. Cells were exposed to 10 ng/ml Stx2 or 3 µg/ml SubAB in growth-arrested conditions for 4, 6 and 24 h. After each period of time, % of necrotic and apoptotic cells were established morphologically by fluorescence microscopy after staining with acridine orange/ethidium bromide (1:1, v/v) and a final concentration of 100 μg/ml [41]. Live cells have normal nuclei staining which present green chromatin with organized structures; apoptotic cells contain condensed or fragmented chromatin (green or orange) and necrotic cells have similar normal nuclei staining as live cells except the chromatin is orange instead of green.

Flow cytometry: Annexin V-FITC/PI double staining assay was used to quantify necrosis and apoptosis. HGEC were seeded into gelatin coated six well cell culture microplates, treated with toxins as described above and subsequently, cells were trypsinized and washed with PBS at pH 7.4. After that, cells were resuspended in binding buffer (0.1 M Hepes, 1.4 M NaCl, 25 mM CaCl_2_) and FITC-conjugated annexin V and PI (propidium iodide) were added. The mixture was incubated for 10 min at room temperature, cells (approximately 50,000 events) were acquired by a Partec model PAS III flow cytometer and data were analyzed by Cyflogic software. The results were interpreted as follows: negative cells for both PI and Annexin-V-FITC staining were considered live cells; PI-negative, Annexin-V-FITC-positive stained cells were considered in early apoptosis; PI-positive, Annexin-V-FITC-positive or PI-positive and Annexin V-negative-stained cells were considered in necrosis.

### Data Analysis

Data are presented as mean ± SEM. Plotting and statistical analysis of data was accomplished using GraphPad Prism 5.0 (GraphPad, USA). Comparisons between values of different groups were performed using one-way ANOVA. Significance was determined using Dunnett’s multiple comparison test. Mann Whitney-test was used for comparison between two groups. Statistical significance was set at *P*< 0.05.

## Results

### Identification of VWF and PECAM- 1 on HGEC

**Figure 1:**
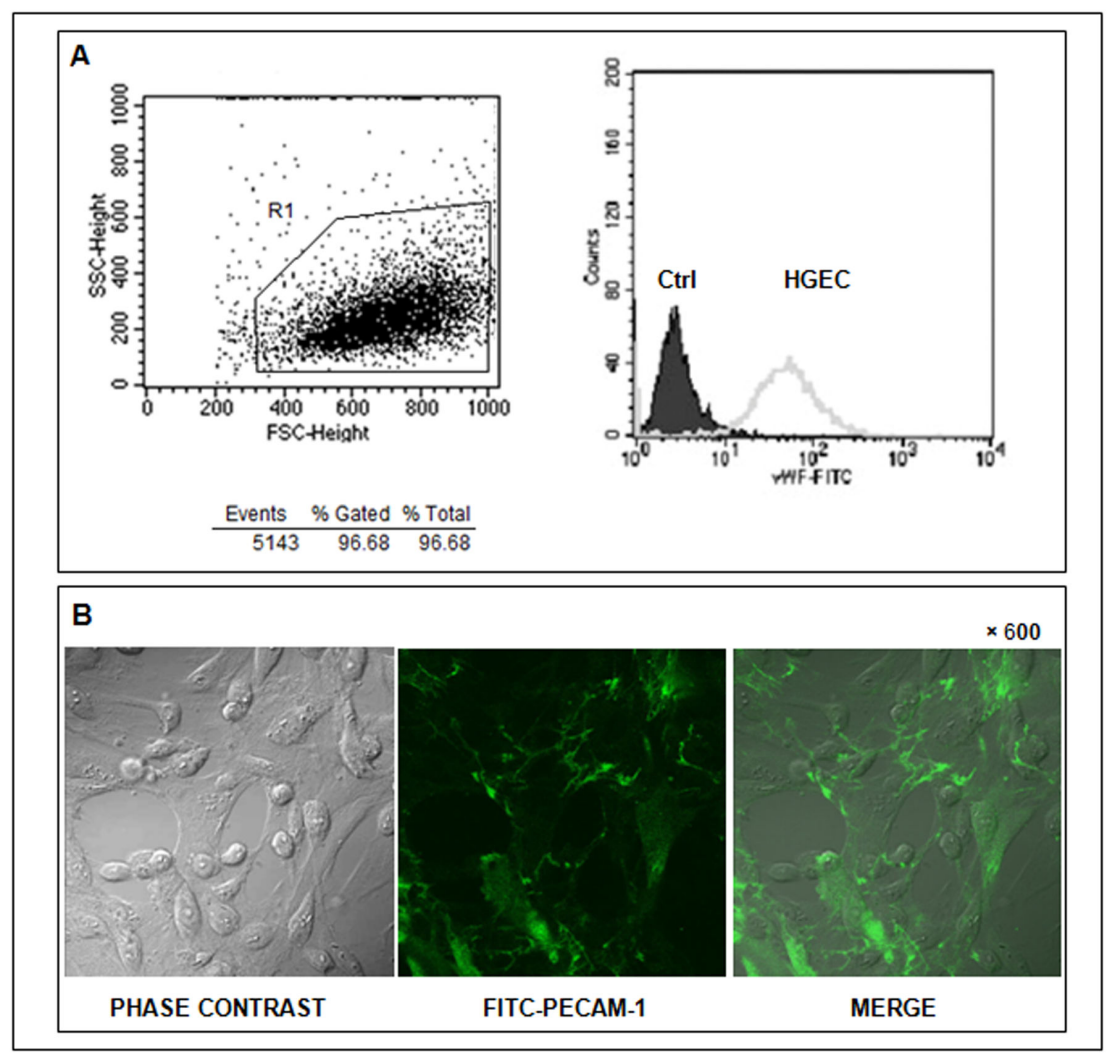
Cells isolated from human pediatric kidneys cortex express characteristic endothelial markers. HGEC seeded in gelatin coated glass coverslips and labeled with either a polyclonal rabbit anti-human VWF and then stained with a goat anti-rabbit FITC or with a FITC-monoclonal mouse anti-human PECAM-1. The expression of these proteins was analyzed by flow cytometry (A) and confocal microscopy (B), respectively.

Cells obtained from human renal glomeruli were cultured and characterized for endothelial phenotype. After the first passage, confluent cells were detached by trypsin treatment and then analyzed by flow cytometry and microscopy. More than 95% of the cells were VWF positive (R1, Figure 1A) when compared to the negative control (cells without staining). The median intensity of fluorescence (MIF) was 60 ± 0.3 *vs* 3 ± 0.2 for VWF and Ctrl, respectively. In addition, these cells also expressed PECAM-1, visualized by confocal microscopy in green fluorescence at cell-cell borders and localized at the plasma membrane (Figure 1B).

### Toxins modified HGEC morphology

**Figure 2:**
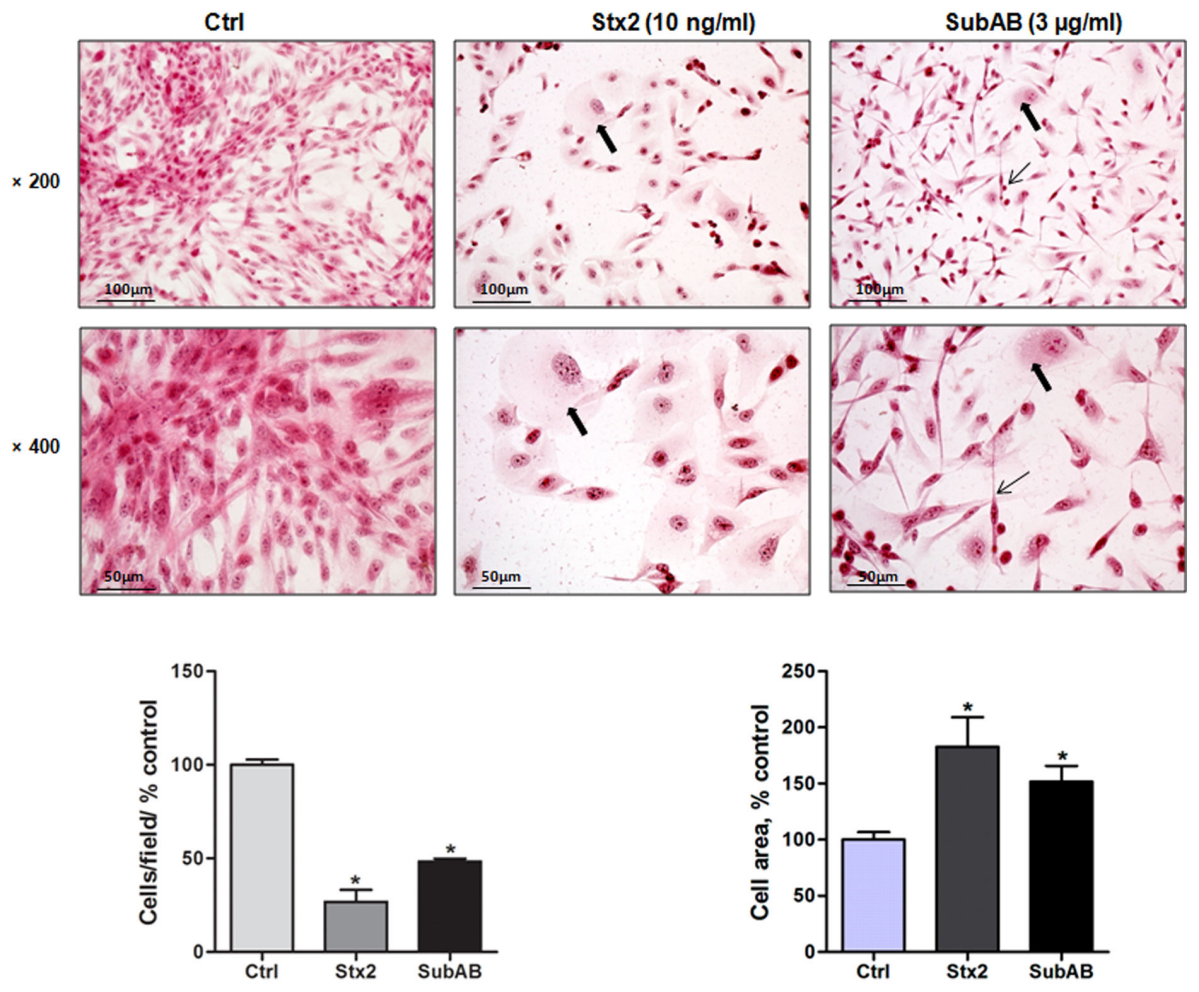
Stx2 and SubAB modify HGEC morphology. Cells were seeded in glass coverslips, treated or not with 10 ng/ml Stx2 or 3 µg/ml SubAB for 24 h and then stained with H and E. Morphology and number of HGEC was analyzed by light microscopy (×200 and ×400). The black arrows indicate the morphological changes as cell swelling (thick arrows) and cell elongation (thin arrows). HGEC areas were measured by using imageJ software. Results are expressed as means ± SEM of three experiments. One hundred percent represents values of cells control, Stx2 or SubAB *vs* Ctrl, *P <0.05.

Cell morphology was analyzed by phase contrast microscopy and additional staining with H&E, so HGEC were incubated under growth-arrested conditions either with Stx2 (10 ng/ml) or SubAB (3 µg/ml) or without toxins for 24 h. Treatment of HGEC with Stx2 resulted in a reduction in viable attached cells (70 ± 6%) and the remaining cells showed an increase of the cell area (90 ± 26%) when compared to control. In addition, SubAB detached fewer cells than Stx2 (50 ± 2%) and the adherent cells showed an elongated shape with increase of the cell area (75 ± 14%) when compared to control, n=3, *P<0.05 (Figure 2).

### HGEC viability decreased after treatment with Stx2 and SubAB toxins

**Figure 3:**
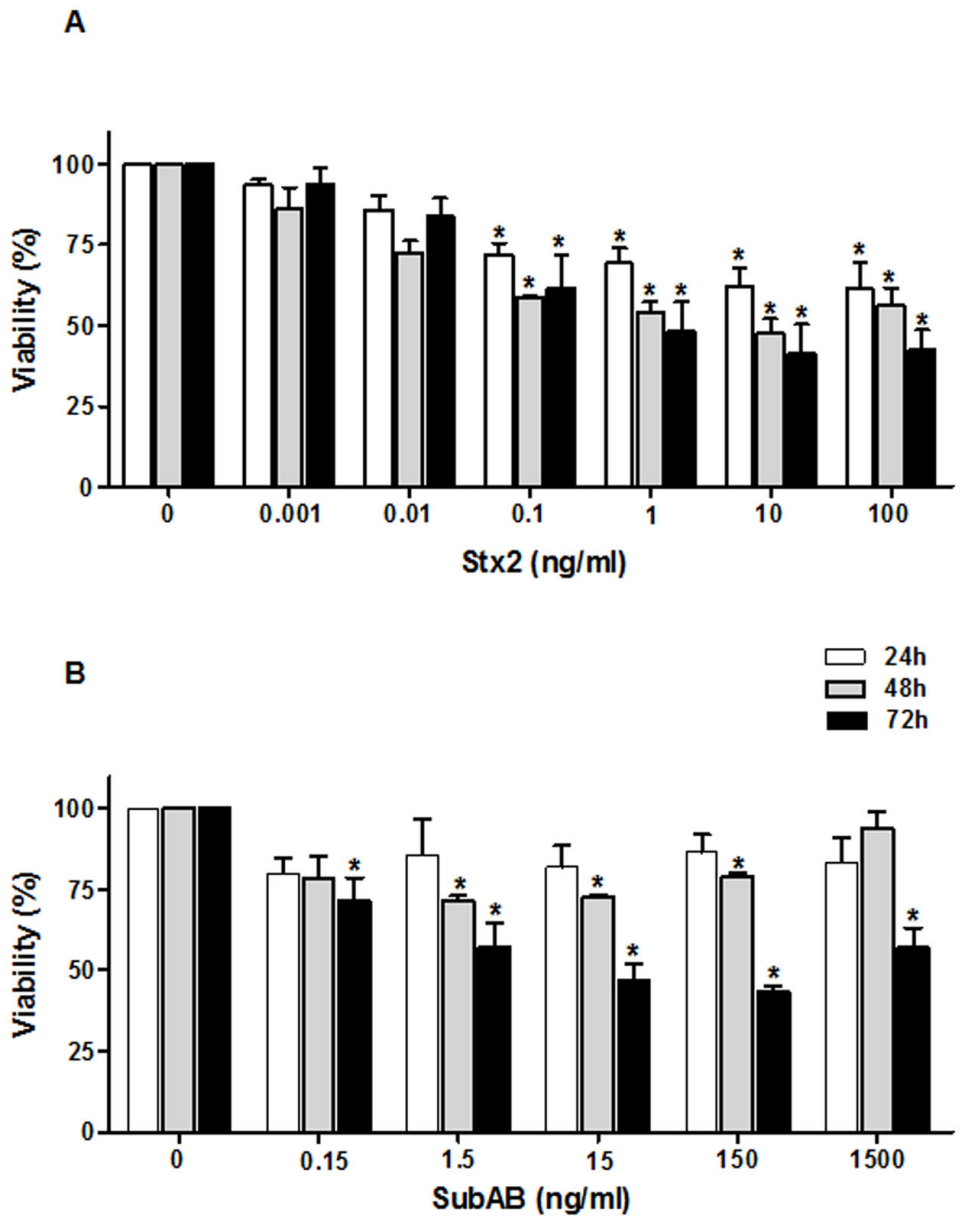
Stx2 and SubAB decrease HGEC viability. Cells placed in 96-well plates were exposed to 0.001 ng/ml to 100 ng/ml Stx2 (A) or 0.15 ng/ml to 1500 ng/ml SubAB (B) in growth-arrested conditions for 24, 48 and 72 h. Then, cells were incubated with neutral red for an additional 1 h at 37 °C in 5% CO_2_. Absorbance of each well was read at 540 nm. One hundred percent represents cells incubated under identical conditions but without toxin treatment (Ctrl). Results are expressed as means ± SEM of five experiments, Stx2 or SubAB *vs* Ctrl, or 24 h *vs* 48 h *vs* 72 h, *P <0.05.

The effects of Stx2 and SubAB on HGEC viability were evaluated at different concentrations and times. A significant decrease in the viability of cells treated with Stx2 was detected when HGEC were incubated with increasing toxin concentrations for 24, 48 and 72 h relative to controls (Figure 3A). Stx2 caused a significant reduction of HGEC viability in a dose-dependent manner from 0.1 ng/ml to 100 ng/ml at all the time points evaluated. After 72 h the CD_50_ was 1 ng/ml (viability %: 48.3 ± 9.1 *vs* control n=5, *P<0.05). SubAB showed to be more cytotoxic at 72 than 48 and 24 h of treatment (Figure 3B, n=5, *P<0.05). After 72 h, a significantly decrease on HGEC viability was observed with concentrations from 0.15 ng/ml to 1500 ng/ml. The range of SubAB concentrations (15 to 150 ng/ml) represents approximately 50% cell lethality after 72 h of incubation. Co-treatment of HGEC with Stx2 and SubAB showed no significant differences in the effects of cytotoxicity compared to those caused by each toxin alone (data not shown).

### Gb3 present on HGEC surface is inhibited by C-9

**Figure 4:**
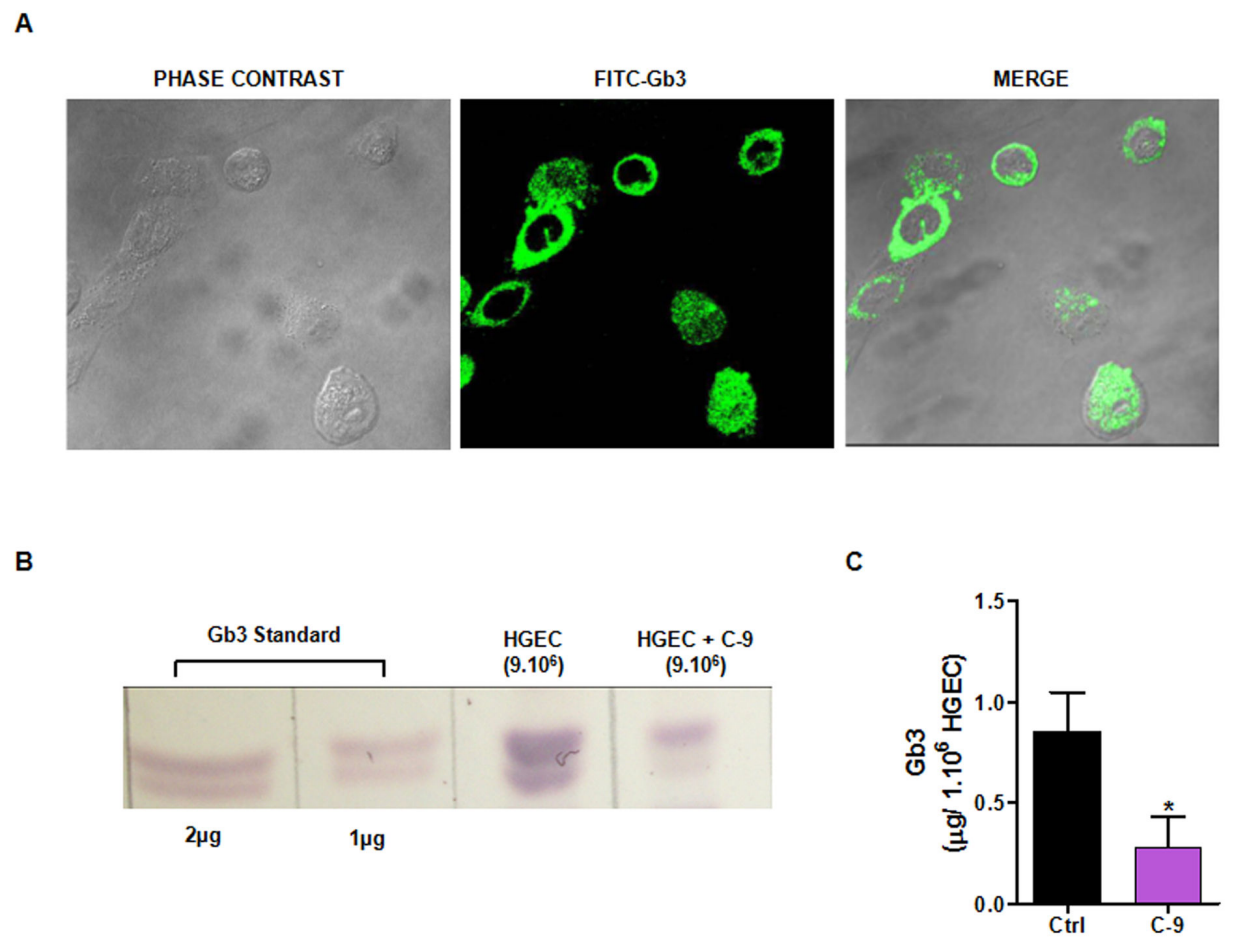
Gb3 is present on HGEC. HGEC seeded in glass coverslips were labeled with an anti-human CD77 conjugated with FITC and visualized by confocal microscopy (×600) (A). Neutral glycolipids extracted from HGEC non-treated (Ctrl) or treated with C-9 (5 µM) for 48 h were subjected to TLC and visualized with orcinol (B). Gb3 was quantified after hydrolysis to galactose by an enzymatic fluorometric method and results are expressed as means ± SEM of three experiments, *P<0.05 (C).

Taking into account the sensitivity of HGEC to Stx2, the presence of Gb3 receptor on HGEC was analyzed by immunofluorescence and TLC. Figure 4A localized Gb3 as a green halo on the surface membrane; it was not detected in the nucleus or mitochondria under optimal antibody concentrations. In addition, neutral glycolipids extracted from HGEC were subjected to TLC and then visualized with orcinol staining. A Gb3 commercial standard (1-2 µg) was used as a positive control. The glycosphingolipids extracted from HGEC (incubated or not with C-9) showed the same pattern of bands as Gb3 receptor standard (Figure 4B). However, a qualitative decrease of Gb3 expression was observed in HGEC treated with 5 µM C-9 (inhibitor of Gb3 synthesis). To confirm these results we quantified the Gb3 on HGEC treated and non-treated with C-9 by an enzymatic fluorometric method after hydrolysis to galactose. As shown in Figure 4C, 1.10^6^ of HGEC contained 0.85 ± 0.20 µg of Gb3 and this concentration decreased after 48 h of C-9 treatment to 0.28 ± 0.15, n=3, *P<0.05.

### C-9 protected HGEC from Stx2 cytotoxic effects

As we demonstrated above, Gb3 receptor is present on HGEC. As well, we found that C-9, a glucosylceramide synthase inhibitor, was able to decrease the Gb3 concentration in these cells. Taking into account these results, we evaluated the effect of Stx2 (10 ng/ml), or SubAB (3 µg/ml) in HGEC previously treated or not with different C-9 concentrations (0.05 µM to 50 µM). After 24 h, the cell viability obtained with Stx2 was 54.0 ± 1.3%, n=4, *P<0.05. When cells were pre-incubated with C-9 for 48 h, followed by Stx2 or SubAB for 24 h, inhibition of Stx2 but not SubAB effects was significantly attenuated in a dose-dependent manner (Table 1). C-9 (50 µM) was cytotoxic after 24 h of treatment (37.0 ± 1.0. *vs* control, n=4, *P<0.05, data not shown).

**Table 1:** C-9 protects HGEC viability from Stx2 toxic effect but not from SubAB.

C-9 (µM)	Viability(%)			Cytotoxicity protection (%)
	C-9, 48 h	C-9 48 h, SubAB 24 h	C-9 48 h, Stx2 24 h	
0	94.0 ± 1.2	77.2 ± 4.5	54.0 ± 1.5	0
0.05	80.1 ± 7.3	75.0 ± 7.2	72.1 ± 4.3*	70
0.5	85.0 ± 3.6	75.2 ± 4.5	87.0 ± 10.0*	100
1	87.5 ± 3.5	73.0 ± 6.3	87.3 ± 7.3*	100
5	83.2 ± 8.6	72.0 ± 7.5	93.0 ± 8.5*	100

* P<0.05, n 4 [C-9 48h, Stx2 24h] *vs* Stx2

### Stx2 and SubAB induced necrosis and apoptosis on HGEC

**Figure 5:**
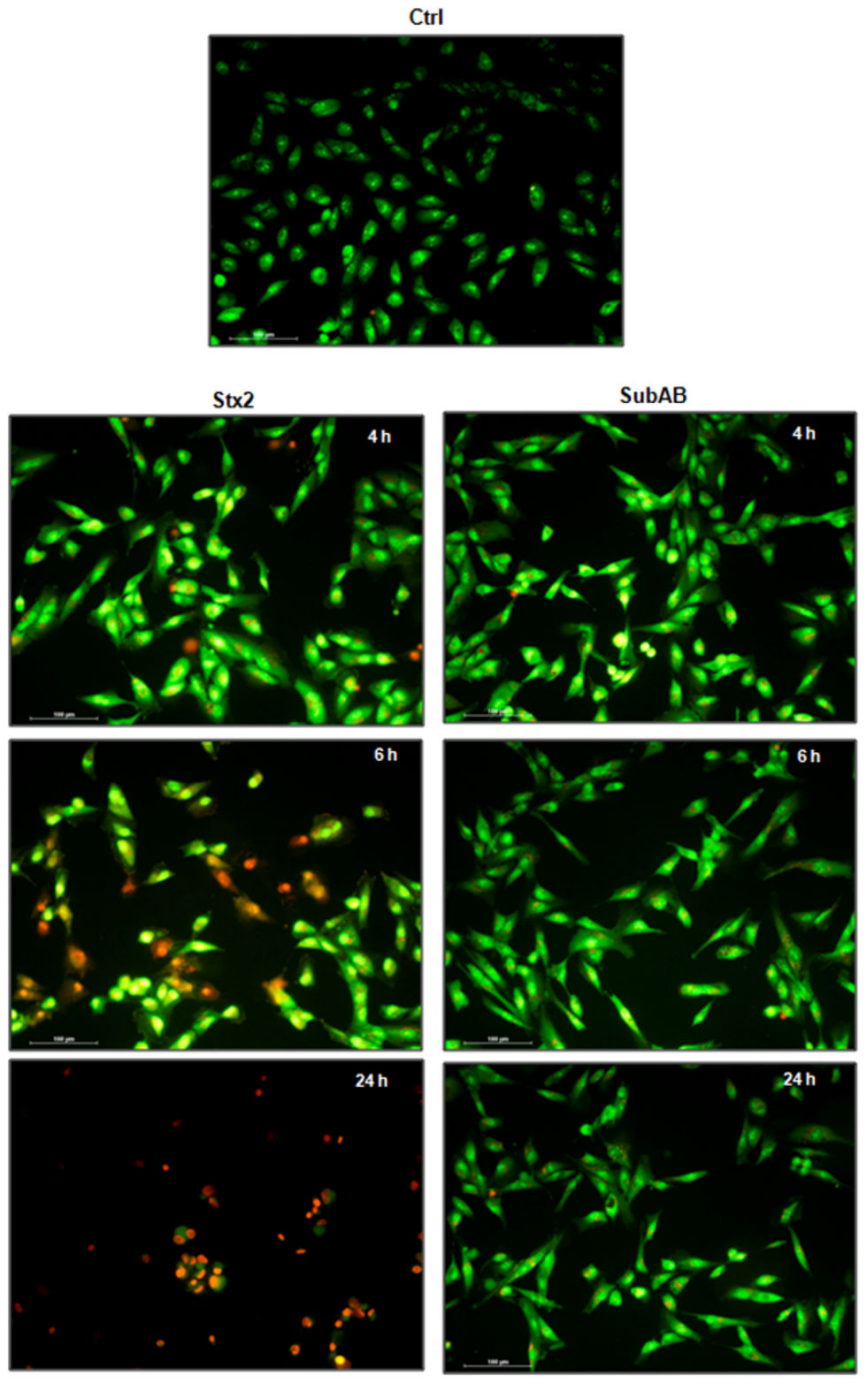
Morphologic changes by Stx2 and SubAB relative to apoptosis and necrosis. HGEC seeded in glass coverslips were exposed or not (Ctrl) to 10 ng/ml Stx2 or 3 µg/ml SubAB in growth-arrested conditions for 4, 6 and 24 h. After each period of time, % of necrotic and apoptotic cells was established morphologically by fluorescence microscopy after staining with acridine orange/ethidium (×200).

**Figure 6:**
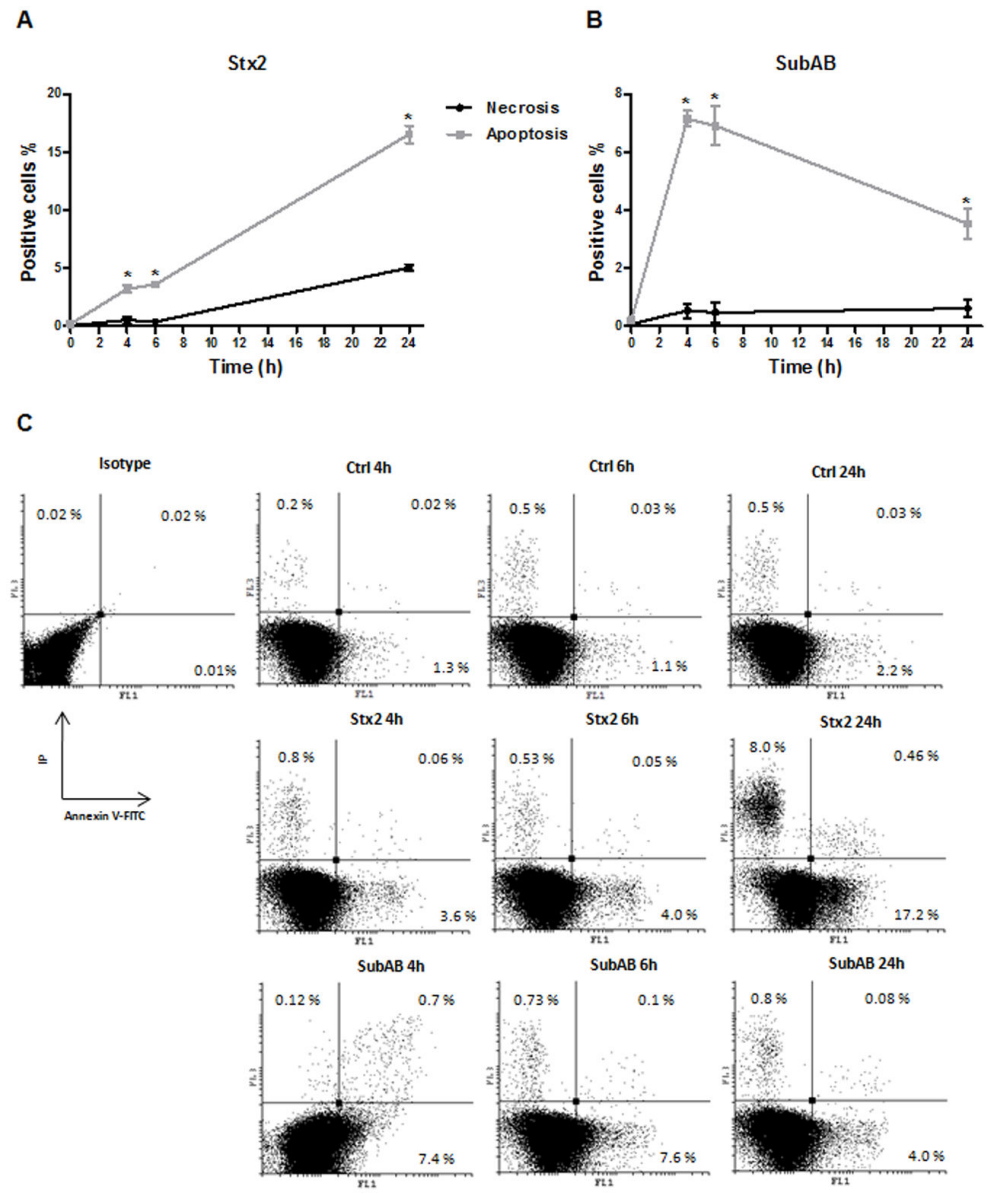
Stx2 and SubAB produce death cell through apoptotic mechanisms. Annexin V-FITC/IP double staining assay was used to quantify necrosis and apoptosis by a flow cytometer. HGEC were treated with 10 ng/ml Stx2 (A) or 3 µg/ml SubAB (B) and labeled with annexin V-FITC/IP for 10 min. Results are expressed as means ± SEM of three experiments. *P<0.05 for necrosis *vs* apoptosis. A representative experiment is shown in panel C.

We then studied the mechanisms of cell death induced by both toxins on HGEC using fluorescence microscopy to analyze cells stained with acridine orange/ethidium bromide and flow cytometry for cells labeled with Annexin V-FITC/IP double staining. The morphologic analysis showed that both toxins increased the apoptosis and necrosis on HGEC. Figure 5 shows apoptosis by condensed chromatin (green and orange) and necrosis by orange chromatin. The results obtained by flow cytometry analysis (Figure 6 A and B) indicate that Stx2 (10 ng/ml) and SubAB (3 µg/ml) caused more apoptosis than necrosis at all times considered (4, 6 and 24 h, n=3; *P<0.05). While Stx2 increased apoptosis in a time-dependent manner, SubAB increased apoptosis at 4 and 6 h but decreased at 24 h. The apoptosis induced by SubAB was higher than Stx2 at 4 and 6 h (4 h: 7.1 ± 0.3 *vs* 3.2 ± 0.3; 6 h: 7.0 ± 0.7 *vs* 3.6 ± 0.3, SubAB *vs* Stx2, n=3, *P<0.05), but lower than Stx2 at 24 h (3.5 ± 0.5 *vs* 16.5 ± 0.74, n=3, *P<0.05). Furthermore, necrosis caused by Stx2 was significantly higher than that induced by SubAB at 24 h. Figure 6C shows a representative experiment.

## Discussion

Primary cultures of human glomerular microvascular endothelial cells are very useful for studying the involvement of toxins that lead to renal failure in HUS. Renal damage has been associated with Stx1 and Stx2, which promote a pro-thrombotic phenotype with lesions in the microvessels in glomeruli [42]. Recently a new toxin, SubAB, has been described that may be involved in HUS pathogenesis [29,43]. In this work, the effects of Stx2 and SubAB on primary cultures of HGEC have been compared. These cells were characterized as endothelial since they expressed VWF and PECAM-1. Both toxins affected the morphology and viability of HGEC after 24 h. This interaction between HGEC and Stx2 or SubAB triggers swelling and endothelial detachment that is coincident with the pathological description of endothelial damage in HUS [44,45]. It is known that endothelial cell viability is dependent on attachment to basement membrane [46]. In consequence, the decrease of HGEC cell viability may be the result of such detachment.

Stx2 but not SubAB reduced HGEC viability in a dose-dependent manner; this could be a consequence of differential toxin receptor distribution and/or density, or other intracellular responses. Our studies have shown that HGEC express Gb3 and the pre-treatment with C-9 protected the cells against Stx2 toxicity. However C-9 did not protect the viability of HGEC from SubAB effects because this toxin binds glycans terminating in Neu5Gc, a glycan distinct from Gb3 [34]. . While the inability of humans to synthesize this monosaccharide has been described and it is incorporated through food products, the HGEC susceptibility to SubAB action could be explained by the presence of these monosaccharides in the FCS [47].

With regard to the intracellular response, apoptosis in microvascular endothelial cells from human renal glomeruli caused by Stx has been documented [48] and induction of apoptosis by SubAB has also been reported for a variety of cell types, including Vero and HeLa cells [30,36]. To analyze these mechanisms, we studied necrosis and apoptosis of HGEC exposed to Stx2 and SubAB. Both toxins caused significantly more apoptosis than necrosis. While Stx2 increased apoptosis in a time-dependent manner, SubAB caused apoptosis only at the shorter treatment times. This result may be due to the two toxins triggering apoptosis by different routes: Stx2 causes apoptosis following protein synthesis inhibition which in turn leads to ER stress, while SubAB causes apoptosis as a consequence of massive ER stress triggered by the cleavage of BIP [32,49].

Relevant to the above *in vitro* data can be the observation that the damage in endothelial cells is amplified in the presence of inflammatory factors such as TNF-α [19] which can be release from monocytes/macrophages in response to Stx [23]. Also relevant may be the potential role of erythrocytes in the development of the microvascular lesion of HUS. It is assumed that the presence of fragmented erythrocytes during HUS is consequence of mechanical fragmentation of these cells while passing through partially occluded capillaries [48]. One study also reported that erythrocyte membranes were affected by oxidative damage during HUS [50], leading to eryptosis [51]. Eryptosis may increase erythrocyte adhesion to vascular endothelium [52] and promote the release of pro-inflammatory cytokines [53] contributing to the thrombotic cascade initiated by Stx direct binding to endothelial cells.

In summary, Stx2 and SubAB were capable of decreasing HGEC viability by endothelial injury similar to that documented in biopsies of HUS patient kidneys. While Stx2 injury appears to be mediated by its specific receptor, Gb3, as evidenced by the inhibitory effects of the Gb3 synthesis inhibitor C-9, SubAB interacts with distinct glycan structures [36], and hence is unaffected by the drug. Our findings suggest for the first time that SubAB cytotoxin can contribute to HUS through HGEC damage characterized by swelling, detachment and finally decrease of cellular viability. In this regard, apoptosis appears to be one of the mechanisms by which this emerging cytotoxin triggers HGEC death.
